# Insights Into Cetacean Immunology: Do Ecological and Biological Factors Make the Difference?

**DOI:** 10.3389/fimmu.2019.01219

**Published:** 2019-05-31

**Authors:** Letizia Marsili, Giovanni Di Guardo, Sandro Mazzariol, Silvia Casini

**Affiliations:** ^1^Department of Physical, Earth and Environmental Sciences, University of Siena, Siena, Italy; ^2^Faculty of Veterinary Medicine, University of Teramo, Teramo, Italy; ^3^Department of Comparative Biomedicine and Food, University of Padua, Padua, Italy

**Keywords:** MICA, cetaceans, OCs, PBDEs, PAHs, MeHg, BPA

## Abstract

The aim of this study was to evaluate the expression of Major histocompatibility complex (MHC) class I chain-related protein A (MICA) in fibroblast cell cultures of cetaceans (skin biopsies of free-ranging specimens and skin samples of freshly stranded cetaceans) by an immunofluorescence technique and to outline possible variations in MICA expression linked to different ecological and biological factors, while also investigating MICA expression after *in vitro* treatments with different contaminants. Free-ranging or stranded specimens of cetaceans were sampled in the Sea of Cortez (Mexico) (*Balaenoptera edeni, Delphinus capensis*, and *Orcinus orca*) and in the Mediterranean Sea (*Balaenoptera physalus, Physeter macrocephalus, Tursiops truncatus*, and *Stenella coeruleoalba*). Cell cultures were treated with an OC mixture, flame retardants, PAHs, MeHg, and BPA. The three species from the Sea of Cortez showed higher basal activity of MICA and lower levels of DDTs and PCBs than the Mediterranean species. A Pearson's linear coefficient equal to −0.45 also confirmed this tendency to have high levels of MICA and low total OC levels. Treatment of cultured fibroblasts with different contaminants mostly resulted in the upregulation of MICA protein expression by at least one treatment dose; downregulation was also found in some species or treatments. MICA alteration indicates a state of stress of the organism and a modification of the immune system's response and can be proposed as a non-invasive immunological marker that can be measured in skin biopsy samples, thus offering a good alternative to blood measurements.

## Introduction

Studies on the immune system of cetaceans have followed one another over the years and focused on endpoints of innate and adaptive immunity, such as the evaluation of cellular and humoral immune responses (lymphocyte transformation assay, natural killer cell activity, phagocytotic activity, and respiratory burst humoral immune responses), insights into cytokines, and acute phase responses, immunophenotyping of cetacean lymphoid cells, morphology, and pathology of lymphoid organs, and immunotoxic effects of environmental contaminants (1–4). Almost all investigations have been conducted on blood or on cetaceans found freshly stranded. In bottlenose dolphin, the possibility exists to work on live specimens, not only with captive specimens but also with wild populations, whereas for other cetacean species, carrying out blood sampling is absolutely unthinkable if we exclude the specimens kept in captivity.

Having an in-depth knowledge not only on how the cetacean immune system is composed but also on how it is modulated in the natural environment according to biological and ecological factors, while also considering the effects deriving from environmental pollution, is important.

The information concerning “immunologic baseline data” in natural populations and the possible alterations and/or modulations linked to internal and external factors are lacking. Stranded organisms can have infections and/or suffer from other disease conditions so that the data found cannot always be considered as baseline. In most of the published studies, the immunotoxic effects of several contaminants or mixtures were investigated only *in vitro*.

In this context of objective limitations, only a few studies have provided information on possible diversity in the immune response, for example, between different species, or within the same species among populations living in different habitats. More information can be found in the literature regarding the differential responses to contaminants after *in vitro* leukocyte exposure.

An interesting study was conducted by Levin et al. (5), in which blood was sampled from belugas (*Delphinapterus leucas*) from the Mystic Aquarium (CT) and from bottlenose dolphins (*Tursiops truncatus*) from the U.S. Bottlenose Dolphin Navy Marine Mammal Program (San Diego, CA). Leukocytes were isolated and treated with different coplanar and non-coplanar polychlorobyphenils (PCBs) in different combinations and in combination with tetrachlorodibenzo(P)dioxins (TCDDs). The results showed a significant reduction in the phagocytic capacity of neutrophils and monocytes in both species, with a markedly greater percentage effect in dolphins compared to belugas. The dolphins, moreover, showed a markedly lower percentage of neutrophils and monocytes even in the untreated samples compared to the belugas. Uncertainties remain regarding basic differences between the two species or differences related to the animals' environmental status (captive/non-captive).

A study by Fair et al. (6) found an upregulation of several blood immune system analytes in wild bottlenose dolphins in comparison with under human care dolphins, allowing to hypothesize that variation in environmental conditions (temperature, nutrition, veterinary care, and pathogen/contaminant exposure) can modulate immune responses.

In most cases, contaminants were found to act as immunosuppressors in cetaceans, although some immune responses were also found to be upregulated after *in vitro* exposure to contaminants.

Ritz and Streibig (7) calculated the adverse effects of different concentrations of PCBs, Hg, methylmercury (MeHg) and Cd on lymphocyte proliferation in belugas and bottlenose dolphins; belugas seem less sensitive to PCBs than bottlenose dolphins but more sensitive to Hg and Cd.

Further immunotoxicological studies on the bottlenose dolphin showed that the treatment concentrations considered environmentally realistic for Hg, Cd, and Pb produced a significant reduction in lymphocyte proliferation, whereas Hg, Al, and Cd treatment resulted in decreased lymphocyte phagocytosis. Chromium did not show any effects on any immune assay at the concentrations tested (8).

In a field study conducted by Schaefer et al. (9) in *Tursiops truncatus* from the coasts of Florida and South Carolina, an inverse relationship was found between the Hg concentrations in blood and skin and the absolute numbers of lymphocytes, eosinophils, and platelets.

Following bottlenose dolphin exposure to perfluorinated compounds (PFCs), Fair et al. (10) found statistically significant positive associations between these contaminants and several immunological parameters: absolute numbers of CD2+ T cells, CD4+ helper T cells, CD19+ immature B cells, CD21+ mature B cells, CD2/CD21 ratio, MHCII+ cells, B cell proliferation, serum IgG1, granulocytic, and monocytic phagocytosis. No effects were found on natural killer (NK) cell activity.

In the same species, treatments with coplanar non-ortho-PCB congeners and butyltins (TNT and DBT) significantly reduced the proliferation response of peripheral blood mononuclear cells (11).

No effect was found on bottlenose dolphin natural killer (NK) cell activity and lymphocyte proliferation (T and B cell) after *in vitro* exposure of peripheral blood leukocytes to environmentally relevant perfluorooctane sulfonates (PFOS) and a penta-PBDE mixture (DE-71) concentrations (12, 13).

As previously stated, we presently have a fairly complete picture of the immune system of cetaceans and its interactions with the main classes of contaminants; however, great limitations in the study of natural populations and the modulation of their immune systems persist and are derived essentially from the typology of sampling and related tests developed and applied to date, which include blood sampling and use of freshly stranded animals. *In vitro* experiments are very useful to understand the dynamics of the interaction(s) between contaminants and the immune system but cannot completely illustrate what happens in wild populations. *In vitro* tests, in fact, cannot describe the whole-organism level processes, such as cell interactions and messenger molecules or chemical exposure, absorption, metabolism, and excretion. An urgent need exists, consequently, to search for new non-invasive tests that use more easily sampled tissues such as skin biopsies, which can provide information comparable to those obtained from blood tests but can be applied to all free-ranging cetacean species and populations, allowing for a high number of samples to be analyzed.

In this paper, we propose the use of the Major Histocompatibility Complex (MHC) Class I Chain-Related gene A (MICA) as a potential biomarker for the cetacean immune system. Information on Human MICA is available in the literature but nothing exists regarding its presence and behavior in cetaceans.

The Human MICA encodes for a 62-Kda cell surface glycoprotein, which is expressed on endothelial, dendritic and epithelial cells; as well as on fibroblasts; and on many neoplastic and virus–infected cells, serving as a target for cellular and humoral immune responses in transformed cells. The level of expression of MICA protein in epithelial tissues is normally low, but upregulation can occur due to several cellular stress stimuli, including heat shock proteins. MICA also functions as a ligand that is recognized by the activating receptor NKG2D, which is expressed on the surface of NK, NKT, CD8+, and TCRγδ+ T cells. Allelic variants of MICA can result in large differences in NKG2D binding. This differential affinity might affect NK cell activation thresholds and T cell modulation in autoimmune diseases and tumors (14, 15). Soluble forms of MICA molecules (sMICA) can also be found, and altered serum levels of sMICA have been reported in multiple health and disease conditions (16).

Zou et al. (17) documented MICA expression on freshly isolated human fibroblasts and a marked decrease when fibroblasts were grown to confluence in culture dishes. By contrast, increased MICA expression was found during the proliferation of fibroblasts, possibly acting as a support for the host response to injury.

Much interest exists in the study of the relationship of MICA with cancer. MICA was found to be expressed on the surface of several neoplastic cells, where it may enhance innate immunity by stimulating NK cells and participating in T cell immunity by costimulation of CD8+lymphocytes. Several tumors can release soluble MICA, and soluble MICA has been reported to inhibit the stimulating pathway mediated by NKG2D.

Cetacean morbilliviruses and papillomaviruses, as well as *Brucella* spp. and *Toxoplasma gondii*, are thought to interfere with population abundance by inducing high mortalities, lowering reproductive success or by synergistically increasing the virulence of other infectious pathogens. Severe cases of lobomycosis and lobomycosis-like disease (LLD) indicate that these infections may contribute to the death of some dolphins. Environmental contamination seems to play a role in these diseases because, as already reported, many pollutants are known as immunosuppressors and can adversely affect the immune status of cetaceans. The cetacean skin is an important tissue district of the immune system, and the MICA protein, which was used in this study as a toxicological stress marker of the immune system, is expressed in fibroblasts.

The aim of this study, in fact, was to evaluate MICA protein expression in fibroblast cell cultures of cetaceans (skin biopsies of free-ranging specimens and skin samples of stranded cetaceans within 2–12 h of death) by an immunofluorescence technique and to outline possible variations in MICA expression linked to differential ecological and biological factors, while also investigating MICA expression after *in vitro* treatments with different contaminants. Free-ranging or stranded specimens of cetaceans were sampled in the Sea of Cortez (Mexico) [Bryde's whale (*B. edeni*), long-beaked common dolphin (*D. capensis*), and killer whale (*O. orca*)] and in the Mediterranean Sea [fin whale (*B. physalus*), sperm whale (*P. microcephalus*), *T. truncatus*, and striped dolphin (*S. coeruleoalba*)], and the cell cultures were treated with organochlorine compound (OC) mixture, flame retardants, polycyclic aromatic hydrocarbons (PAHs), MeHg, and bisphenol A (BPA).

## Materials and Methods

### Sampling Methods

#### Free-Ranging Cetaceans

Samples of skin biopsies (epidermis, dermis, and blubber) were obtained from free-ranging specimens of long-beaked common dolphin (MDC12) and striped dolphin (STG96) using an aluminum pole armed with biopsy tips (0.7 cm ø, 3.0 cm length), while skin biopsies from free-ranging specimens of Bryde's whale (MBE3), killer whale (MOO12), sperm whale (PMAs1 and PMAs2), fin whale (BPT1), and bottlenose dolphin (TTAs1) were obtained using a Barnett Wildcat II crossbow with a 150-pound test bow, using a biopsy dart with modified stainless steel collecting tip (0.9 cm ø, 4.0 cm length). Biopsy samples were taken in the dorsal area near the dorsal fin, with CITES authorization (CITES Nat. IT025IS, Int. CITES IT 007), in the Sea of Cortez (MDC12, MBE3, and MOO12) and Mediterranean Sea (PMAs1, PMAs2, BPT1, TTAs1, and STG96). A little biopsy fragment was immediately stored in cell medium for cell cultures.

#### Stranded Cetaceans

Skin tissue of stranded cetaceans (dead for only 2–12 h) was obtained from specimens found dead along the Italian coastline in the period 2005–2009. Samples were taken from beneath the dorsal fin of stranded specimens of fin whale (RT25) and striped dolphin (RT17 and RT23) and immediately placed in cell medium.

#### Sex Identification

Sex determination in cetaceans was performed by genetic investigations according to Berubè and Palsboll (18). The sex of specimens was as follows: MDC12 female (F), STG96 male (M), MBE3 (F), MOO12 (M), PMAs1 (M), PMAs2 (M), TTAs1 (F), RT25 (M), (BPT1 (F), RT23 (F), and RT17 (M).

### Fibroblast Cell Cultures

The development of a non-invasive sampling method for obtaining viable tissue samples for cell cultures from skin biopsies of free-ranging and stranded cetaceans was described by Marsili et al. (19). Successful cell cultures were obtained from all the specimens.

After the animals were sampled, each skin sample was stored in sterile medium MEM Eagle Earle's salts w/L-glutamine and sodium bicarbonate + 10% gamma irradiated fetal calf serum + 1% MEM non-essential amino acids (NEAA) solution 100x + 1% Penicillin/Streptomycin 100x + 0.1% Amphotericin B 100x at room temperature and was processed within 24 h of collection. In the laboratory, each sample was washed with Earle's balanced salt solution (EBSS) containing antibiotic (Penicillin/Streptomycin 100x) and antimycotic (Amphotericin B 100x) solutions. All specimens were handled under strict sterility conditions. First, the collected tissue was cut into small pieces with curved surgical scissors, placed in 30-mm Petri dishes and incubated with Trypsin-EDTA solution 1x for 15 min at 37°C. The biopsy fragments were washed again and then placed in Falcon 25 flasks, moistened with medium. After 24 h at 37°C in an incubator with 5% CO_2_, the cultures were covered with 1 ml of medium. Half of the culture medium was replaced every 48 h with fresh medium.

### Indirect Immunofluorescence Technique

Fibroblast cell cultures (third generation) were subjected to this experimental protocol for 48 h. The preliminary test that we conducted concerned the treatment of fibroblasts with an inducer and a repressor of the immune system. We used cyclosporine A (CsA), a drug belonging to the category of immunosuppressants, along with β-glucan, a polysaccharide known to increase immune system response. For the main research the different cell lines were exposed as follows: (1) to a mixture of organochlorines (Arochlor 1260, pp′-DDT and pp′-DDE), solubilized in dimethyl sulfoxide (DMSO) (0.05%) (named OCs), at three doses: 0.01, 0.1, and 1 μg/ml, plus a DMSO (0.05%) control; (2) to a mixture of benzo(a)pyrene (1 mM) and beta-naphthoflavone (20 mM), solubilized in acetone (0.1%) (named PAHs), at three doses: C = (0.5 μM BaP + 10 μM BnF), B = (2.5 μM BaP + 50 μM BnF), and A = (12.5 μM BaP + 250 μM BnF), plus an acetone (0.1%) control; (3) to a mixture containing 27 polybrominated diphenyl ethers (PBDEs), from mono- to deca-brominated (BDE-MXE), solubilized in nonane (0.01 μg/ml) (named PBDEs) at three doses: 0.01, 0.05, and 0.1 μg/ml, plus a nonane (0.01 μg/ml) chemical control; 4) to bisphenol A (BPA) solubilized in ethanol (0.1%) (named BPA), at four doses: 0.1, 1, 10, and 100 μg/ml, plus an ethanol (0.1%) control; and 5) to hydrosoluble and therefore solubilized in cultured medium methylmercury (MeHg), at four doses: 0.01, 0.1, 1, and 10 μM.

We applied immunofluorescence to fibroblast cell cultures for a qualitative and semiquantitative analysis of target protein MICA. After fixing and extraction with methanol and acetone at −20°C, we conducted a first reaction with the primary polyclonal antibody (Ab) (rabbit polyclonal anti-MICA Ab; Abcam), the cells were treated with the secondary Ab (Alexa Fluor 568 rabbit anti-goat IgG (H + L) for MICA) and labeled with red-fluorescent Alexa Fluor dye. Immunofluorescence was quantified with a specially designed Olympus Soft Imaging Systems macro, *DetectIntZ*, which works with the image acquisition, processing and analysis system, *analySIS*^*B*^ (Olympus) (20). The image analysis procedure has the objective of quantifying, with an adimensional index generated for this purpose, the amount of Alexa Fluor localized on the cytoplasmic membrane of the various cell preparations. The cells under study were imaged using DAPI, and this image was presented to the operator for threshold selection of cytoplasmatic and nuclear regions of interest (ROI) across the field. The procedure then utilized these ROI to measure the fluorescence intensity with Alexa Fluor of the different cell samples and the results were summarized in a worksheet. The system generated index values that are unitless until compared with other units, such as number of cells to obtain mean fluorescence per cell or the area in which it was calculated to obtain the mean fluorescence per mm^2^. Images were all obtained with a magnification of 20X, a calibration of 0.65 μm/pixel and a resolution of 1,360 × 1,024 x 8 pixel. Exposure times were fixed while reading MICA expression for each treatment. A series of images of each slide was acquired so that a minimum of 250 cells/slide could be counted. The total fluorescence revealed by the program was then divided by the number of cells to obtain an arbitrary unity of fluorescence (AUF) per cell. Several slides for MICA were made for each culture: one was a blank (cells treated only with primary and secondary antibodies), one was a secondary blank (cells treated only with the secondary antibody), one was a chemical blank (cells treated with contaminant carrier), and two were for each treatment dose of all contaminants. The former two blank samples enabled the natural presence of the target proteins in cultured fibroblasts to be checked, while the secondary blank enabled validation of the dose of secondary Abs without cross reaction, as the primary Ab was absent.

### Organochlorine Analysis

Analyses for DDTs and PCBs were performed according to methods recommended by the U.S. Environmental Protection Agency (EPA) 8081/8082, with modifications (21, 22). The analytical method used was High Resolution Capillary Gas Chromatography with an Agilent 6890N and a 63Ni ECD and an SBP-5 bonded phase capillary column (30 m long, 0.2 mm i.d.). The carrier gas was nitrogen, with a head pressure of 15.5 psi (splitting ratio 50/1). The scavenger gas was argon/methane (95/5) at 40 ml/min. The oven temperature was 100°C for the first 10 min, after which it was increased to 280°C at 5°C/min. Injector and detector temperatures were 200 and 280°C, respectively. The extracted organic material (EOM%) from the freeze-dried samples was calculated in all samples. Capillary gas chromatography revealed op′- and pp′- isomers of DDT and its derivatives DDD and DDE and revealed ~30 PCB congeners. Total PCBs were quantified as the sum of all congeners. These congeners represented 80% of the total peak area of the PCBs in the samples. Total DDT was calculated as the sum of op-DDT, pp′-DDT, op′-DDD, pp′-DDD, op′-DDE and pp′-DDE. The results were expressed in nanograms per gram of lipid weight (ng/g l.w.).

### Statistical Analysis

Shapiro-Wilks test has been used to evaluate if the population is normally distributed (*p* > 0.05). Findings showed that all the investigated groups were non-normal distributed. Descriptive statistics (mean, standard deviation, minimum, and maximum) was used to summarize the data. The Spearman's rank correlation coefficient (*r*-Spearman) was instead used in order to measure the degree of association between This indicator ranges from +1 to −1. A value of 0 indicates that no association exists between the two variables. A value greater/lower than 0 indicates a positive/negative association between the variables investigated. Finally, the non-parametric tests of Kruskal-Wallis (*p* < 0.05) and Kolmogorov-Smirnov (*p* < 0.05) were used to determine if the levels of MICA, DDTs, and PCBs significantly differ between specimens and if the levels of MICA in the cells significantly differ between doses and type of treatments.

## Results and Discussion

### Basal Levels of MICA in Different Species

**Figure 1:**
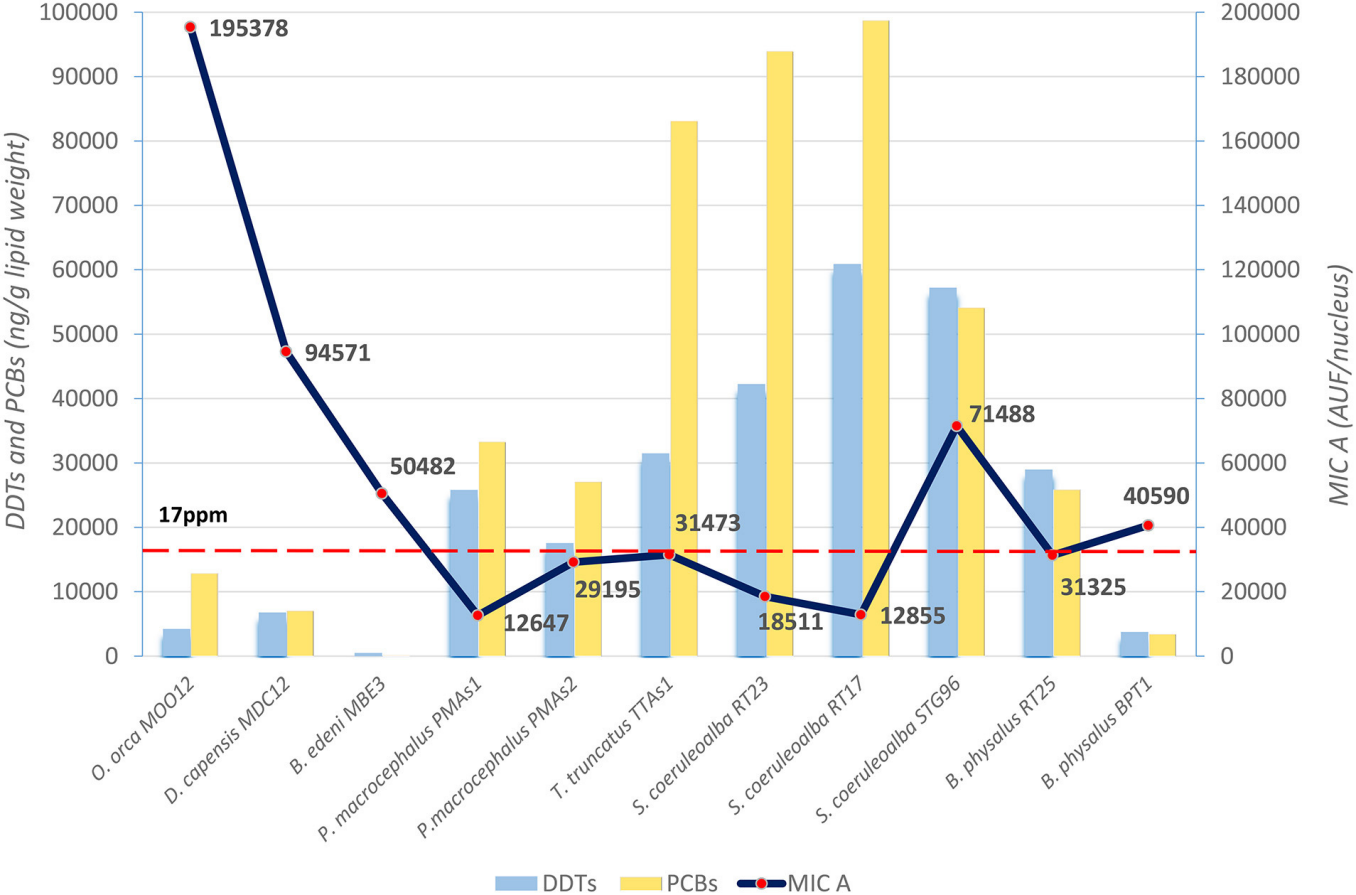
Basal levels of immunofluorescence (AUF/nucleus) of MICA (line) in fibroblast cells and mean levels of DDTs and PCBs (ng/g lipid weight) (histograms) in blubber *of B. edeni, D. capensis, O. orca, P. macrocephalus, T. truncatus, S. coeruleoalba, and B. physalus*. The red dotted line represents the PCB threshold (17 ppm l.w.) indicated to produce deleterious effects in marine mammals.

The basal level of MICA, evaluated with immunofluorescence technique in the fibroblasts of different cetacean species, is the first important result to indicate the immune status of the specimens sampled in different areas. In particular, these data were compared to DDT and PCB levels detected in the blubber samples of the same specimens. The mean values, expressed as immunofluorescence for cell (AUF/nucleus) for MICA and as ng/g lipid weight for OCs, are presented in Figure 1.

**Figure 2:**
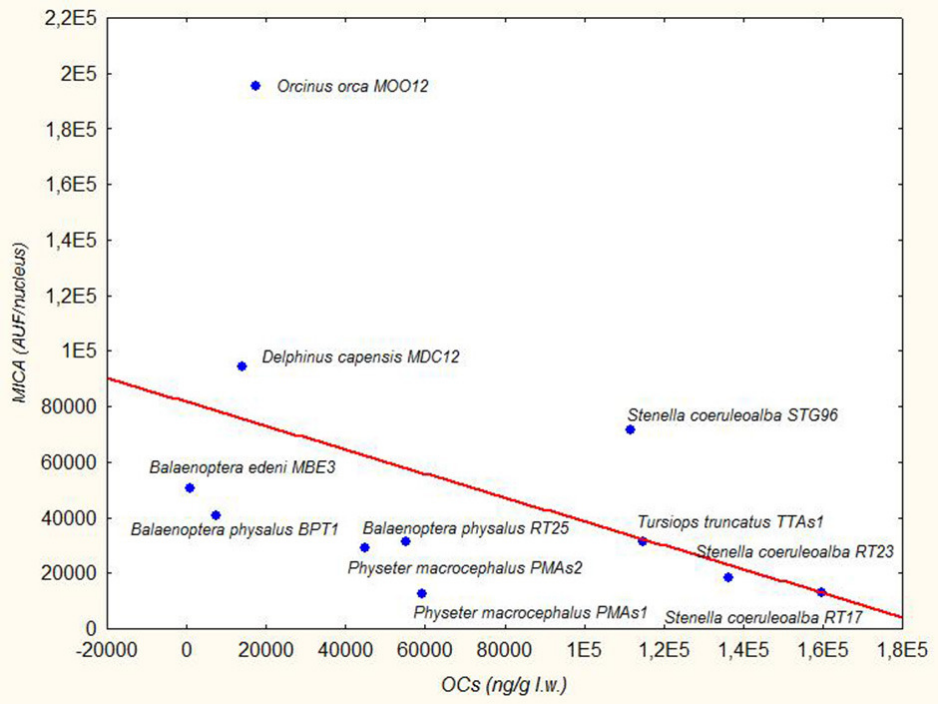
Trend Line (Spearman's rank correlation coefficient *r* = −0.59) between MICA (AUF/nucleus) in fibroblast cells and OCs (ng/g lipid weight) in blubber of *B. edeni, D. capensis, O. orca, P. macrocephalus, T. truncatus, S. coeruleoalba, and B. physalus*.

The three species from the Sea of Cortez (*B. edeni, D. capensis*, and *O. orca*) exhibited a basal activity of MICA higher than the Mediterranean species, except for the free-ranging striped dolphin STG96, and correspondingly lower levels of DDTs and PCBs. Despite the low total sample number, a Spearman's rank correlation coefficient equal to −0.59 also confirmed this tendency for high levels of MICA and low total OC levels (Figure 2). In fact, in the guidelines to interpreting Spearman's rank correlation coefficient, this association is medium. Interestingly, in the Mexican species, the PCB levels did not exceed the estimated toxicity threshold (17 mg/kg l.w.) for deleterious health effects set by Jepson et al. (23) and Kannan et al. (24) (Figure 1); this indicates that, in this area the toxicological risk for these marine mammals is low, regardless of their position in the trophic chain. Instead, the basal activity of MICA in species of the Sea of Cortez seems to be related to their different diets, with an enhanced activity parallel to the increase of their trophic level, while noting that *B. edeni* feeds mainly on blue fish and is not strictly plankton-eating as *B. physalus* is in the Mediterranean Sea. However, the total OC levels of *B. edeni* were the lowest, showing that plankton intake is not negligible in the diet. In the Sea of Cortez, the specimens had been sampled while free-ranging, by skin biopsy, so they were all supposedly healthy; consequently, the differences both in the accumulation and in the immune response were most likely linked to ecological and biological factors and not to a more or less compromised health condition, as in the case of stranded specimens. In the Mediterranean Sea, instead, three samples came from specimens found stranded alive and then died (*B. physalus* RT25 and *S. coeruleoalba* RT17 and RT23), while five came from free-ranging specimens (*P. microcephalus* PMAs1 and PMAs2, *T. truncatus* TTAs1, *B. physalus* BPT1, and *S. coeruleoalba* STG96). Regardless of the type of sampling (free-ranging or stranded), the health condition, the position in the trophic chain, and the sex, all the herein investigated specimens had consistent tissue levels of both DDTs and PCBs. The latter xenobiotics far exceeded the toxicity threshold of 17 ppm, except for the free-ranging fin whale BPT1, and the MICA response was globally much lower than in the Sea of Cortez species.

Since MICA has a non-parametric distribution, evaluated with the Shapiro-Wilks test (*p* < 0.00156), a non-parametric statistical analysis was used to compare the different variables. Using the Kruskal-Wallis test and putting the data together according to the sampling area, without considering the single species, we verified that MICA expression was significantly different (*p* = 0.0247) between the Sea of Cortez and the Mediterranean Sea samples. The same occurred with the levels of DDTs and PCBs (*p* = 0.0412).

Based upon the results of the present study, we can affirm that the environment in which the specimens live and, accordingly, the anthropogenic stress “magnitude” to which they are subjected, are key determinants in MICA protein-based immune responses. It is well-known that the Mediterranean Sea is highly contaminated by several anthropogenic activities, such as coastal urbanization and industrialization, oil tanker traffic and general maritime transport, fisheries, and agricultural waste (25). Sea of Cortez instead is considered close to a pristine environment, with a low anthropogenic impact (26). Therefore, it can be also inferred that the lower the anthropogenic stress of the specimens is, the higher is the basal activity of MICA.

Regarding the Mediterranean species, the two fin whale specimens had the lowest OC levels related to other species, and this is in line with what we have always found in this basin, depending on the different trophic position (21, 27). However, the *B. physalus* RT25 concentrations were very high compared to those found in *B. physalus* BPT1 (~7 times for the DDTs and PCBs) and in other Mediterranean fin whale specimens, both stranded and free-ranging (25). The mysticete RT25, found stranded in January 2011 along the Tyrrhenian coast of Italy, showed an unprecedented coinfection by *Dolphin Morbillivirus* (DMV) and *Toxoplasma gondii*, together with high OC pollutant concentrations, with special reference to DDTs (28). Therefore, the basal activity of MICA might have been higher than that of a free-ranging fin whale as a function of a response to these multiple stress factors Instead, the free-ranging fin whale BPT1 had a higher MICA value, with considerably lower OC xenobiotic levels. Within such context, it is of interest that the hepatitis B virus (HBV), for example, suppresses the expression of MICA/B on hepatoma cells through the upregulation of transcription factors GATA2 and GATA3 to escape from NK cell immune surveillance (29), and a similar pathogenetic mechanism could also be shared by DMV and *T. gondii*. In addition, for the striped dolphin specimens, we obtained comparable results: RT17 and RT23, both stranded animals, had PCB levels higher than free-ranging STG96, but the opposite was true for MICA. Furthermore, the RT23 individual was also infected by DMV. The other specimens sampled by biopsy, belonging to bottlenose dolphin (TTA1) and sperm whale (PMAs1 and PMAs2) species, had MICA levels like the stranded striped dolphin and fin whale specimens. An explanation could be linked also to interspecies differences or to sex or age variability. In this respect, a non-parametric statistical test (Kolmogorov-Smirnov) showed that no significant differences existed between males and females in MICA levels, without considering the sampling area and the species. In the striped dolphin group, however, we had two specimens of different sex that were both stranded, with an adult and almost certainly sexually mature (190 cm) individual (RT23) and a male subadult (168 cm) (RT17). In these specimens, at similar tissue concentrations of OC pollutants, a similar level of MICA expression was found. All that we have highlighted with these results makes it difficult to explain if the activity of MICA has an upregulation or a downregulation with a toxicological stress. What we know is that, in humans (*Homo sapiens sapiens*), where it is most studied, MICA protein has a low level of expression in healthy epithelial tissues but is upregulated in many tumors (30–32) and under various cellular stress stimuli, including heat shock proteins, DNA damage, and viral infections (33–36). MICA also functions as a ligand recognized by the activating receptor Natural Killer Group 2D (NKG2D), which is expressed on the surface of Natural Killer (NK) cells, Natural Killer T cells (NKT), CD8+ T cells (often called cytotoxic T lymphocytes, or CTLs) and T-cell receptor (TCR)γδ+ T cells. McGilvray et al. (37) reported that NKG2D ligands were highly expressed in lymph node metastasis of stage I colorectal cancer samples, but they were expressed in lower amounts in Stage II, III, or IV tumors. A higher expression of MICA in colorectal cancer patients was associated with a good prognosis (38). MICA molecules exist also in soluble forms (sMICA) and altered serum levels of sMICA have been reported in multiple health and disease conditions. For example, in the case of hepatitis C virus (HCV)-associated hepatocellular carcinoma (HCC) the control serum had soluble MICA values ≤ 5 pg/ml, whereas the serum from diseased patients had values ≥ 5 pg/ml (39). The higher level of sMICA in the blood serum from HCV-infected, HCC-affected patients highlighted the possible role of MICA as a tumor suppressor. However, the elevation of serum sMICA was shown to be associated with a poor prognosis in various cancer patients (40–43), thereby emphasizing once again the difficulty of interpreting the quantitative response. Nothing is known about the “stress” caused by environmental contaminants, although NKG2D expression has also been shown to be regulated by estradiol (44). Endometrial cells exposed to estradiol upregulated MICA protein expression (45). In this respect, the MICA promoter contains an estrogen receptor response element, suggesting that estrogens may increase MICA expression through transcriptional regulation. Consequently, provided that many of the xenobiotics accumulated by these marine mammals are known as endocrine-disrupting chemicals (EDCs), they could exert biologic activities similar to that of estradiol.

Since MICA expression has been extensively investigated in human studies, a comparison of the basal activity of MICA in cetacean fibroblasts with those of a human biopsy carried out for medical investigations was also deemed of interest. In human fibroblasts, the basal activity of MICA resulted in 11.863 AUF/nucleus and therefore was lower than that of all the herein investigated cetacean specimens. In the biomedical literature, no studies on MICA expression in human fibroblasts have been made available before the present investigation, that was carried out by means of an indirect immunofluorescence technique. Unfortunately, no subcutaneous adipose tissue was sampled in the human biopsy, so that we cannot correlate the levels of DDTs and PCBs with the values of MICA. Regardless of this, we can still infer that the levels of MICA in human fibroblasts are lower than those of all investigated cetacean species but very similar to those of the sperm whale PMAs1 and the striped dolphin RT17 and generally to most of the Mediterranean specimens.

However, given that even in humans, as already mentioned above, whether MICA showed an upregulation or a downregulation in the presence of a “generic” stress(or), is difficult to determine.

The results of each specimen whose cells were treated with the two compounds are expressed as index numbers (AUF Treatment: AUF BA = X Index Number: 100) and shown in Table 1. The sample named BA is the blank or “basal activity sample,” which is the cultured fibroblasts treated only with primary and secondary antibodies. The doses applied to induce and repress expression were established according to the literature (46–51).

**Table 1:** Immunofluorescence of MICA, expressed as index numbers relative to the blank (BA), revealed in cultured fibroblasts of different cetacean species and humans treated with the immune response activator (β-glucan), and suppressor (Cyclosporine A).

	**BA**	**Inducer** **500 μg/ml**	**Repressor** **0.8 μg/ml**	**Repressor** **80 μg/ml**
MOO12 (*O. orca*)	100	96	55	143
TTAs1 (*T. truncatus)*	100	61	69	66
RT23 (*S. coeruleoalba*)	100	95	112	All cells died
RT25 (*B. physalus)*	100	56	90	61
MA1 *(Homo sapiens)*	100	9	70	All cells died

The results were quite contradictory: in killer whales, in fact, MICA showed a downregulation with both the inducer and the lower dose of the repressor but upregulation with the higher dose of repressor; in striped dolphins, by contrast, the upregulation was already present at the lower dose of the repressor, whereas at the highest dose, we observed a complete cell death, with this same effects also occurring in humans, but with both the inductor and the low-dose repressor, downregulation occurs; finally, in bottlenose dolphins and in fin whales, downregulation occurred with all the treatments. Most likely, the choice of the two compounds, known to have such capabilities in relation to the immune system but not specifically in respect to MICA, should be reassessed.

### MICA in Different Species After Treatment With OCs, PBDEs, PAHs, BPA, and MeHg

After the basal activity of the different specimens had been evaluated, trying to evaluate how much MICA was modified following treatment with some of the main environmental contaminants with which these animals come into contact in their living environment was deemed of interest. Since most of these toxicants are not water-soluble and therefore need a carrier (for example, DMSO, or nonane) to treat the fibroblasts, the basal activity with which the different treatments were compared is relative to that given by the carrier. This way of proceeding is correct because upregulation of MICA very often had already occurred with the solvent alone. The toxicity of different solvents to cells was previously demonstrated in other studies (20, 52).

**Figure 3:**
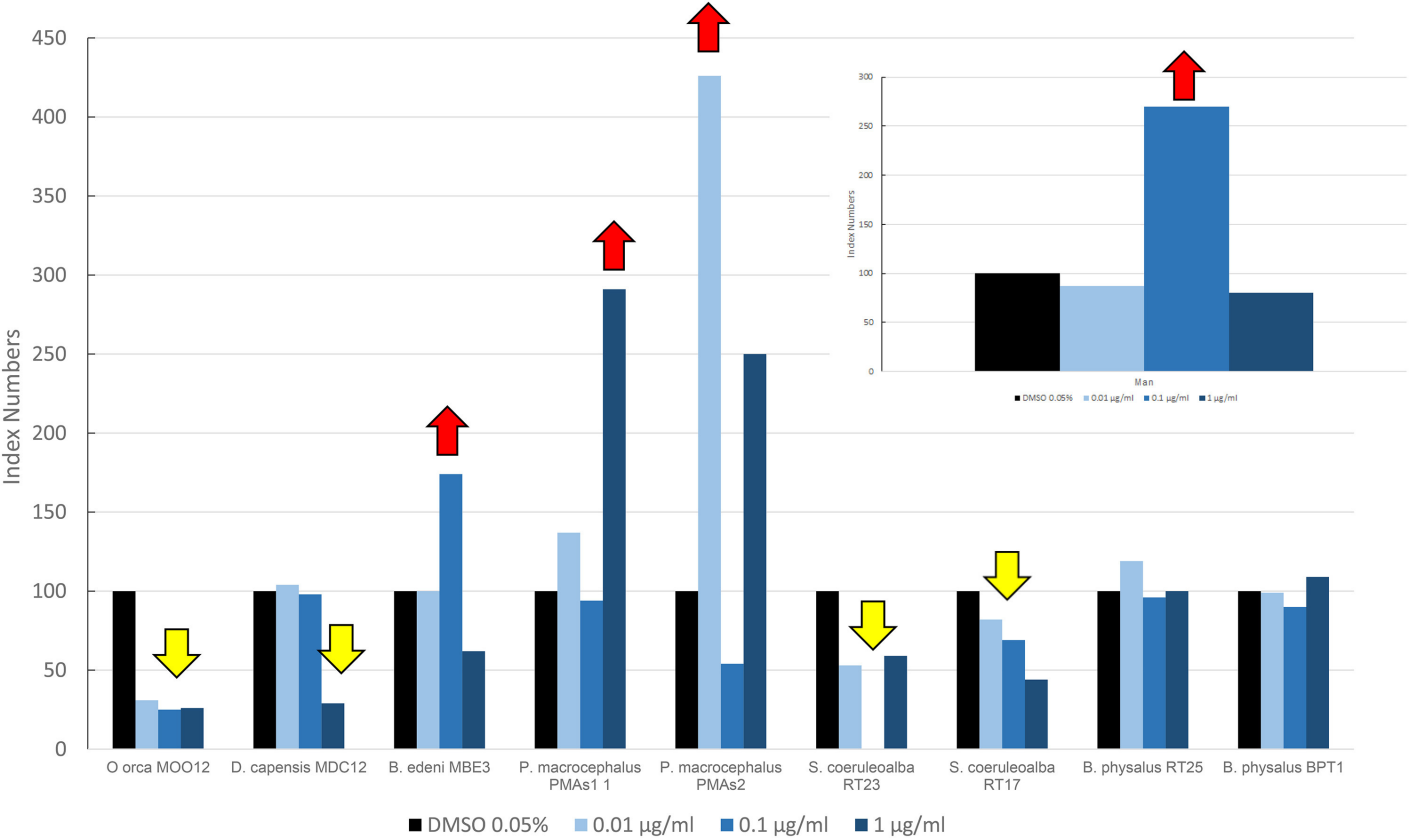
Mean values of immunofluorescence of MICA expressed as index numbers respect to the solvent control (DMSO) revealed in cultured fibroblasts of different cetacean species and in man treated with OCs. Red arrows indicate a significant upregulation respect to DMSO control, yellow arrow a significant downregulation.

In Figure 3, we reported the results of the mean levels of immunofluorescence of MICA, which was revealed in cultured fibroblasts of different species treated with OCs and expressed as index numbers relative to the solvent control (DMSO). According to Figure 3, the response was very different between the species and at different doses: killer whale and striped dolphin specimens showed no increase in MICA expression at any dose; in the long-beaked common dolphin, the MICA level remained unchanged until the highest treatment, where we had a downregulation; the two fin whale specimens showed neither upregulation nor downregulation at any dose; and only in the case of the sperm whale and Bryde's whale that we assisted we observed a clear upregulation effect on MICA due to OCs, compared to DMSO. The two specimens of sperm whale showed the same response curve, while the Bryde's whale had an equal response to that of man, that is, a bell-shaped response.

**Figure 4:**
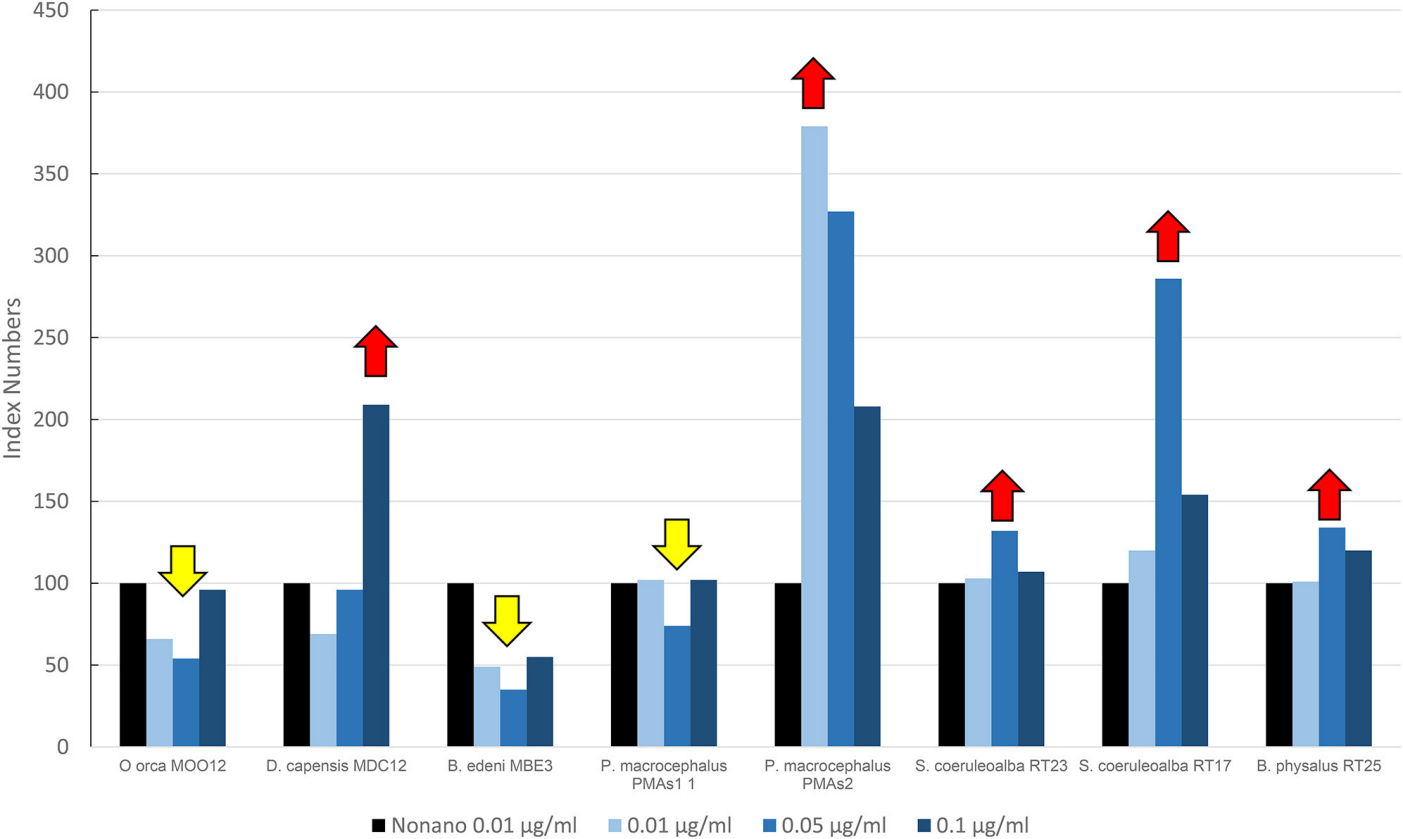
Mean values of immunofluorescence of MICA expressed as index numbers relative to the solvent control (nonane) revealed in cultured fibroblasts of different cetacean species treated with PBDEs. Red arrows indicate a significant upregulation respect to nonano control, yellow arrow a significant downregulation.

Figure 4 shows results of the mean levels of immunofluorescence of MICA, revealed in cultured fibroblasts of different cetacean species treated with the PBDEs, which are expressed as index numbers relative to the solvent control (nonane). In the sperm whale (PMAs2), striped dolphin, and fin whale specimens, the highest response of MICA was the one related to the flame retardant treatment, with a bell-shaped response in all species; the long-beaked common dolphin showed an upregulation at the highest dose; a discontinuous response was shown by sperm whale (PMAS1), whereas Bryde's whale and the killer whale showed a downregulation. Additionally, in this case, as for the OCs, the two striped dolphins responded in the same way, even if the flame retardants lead to upregulation and the OCs to downregulation.

**Figure 5:**
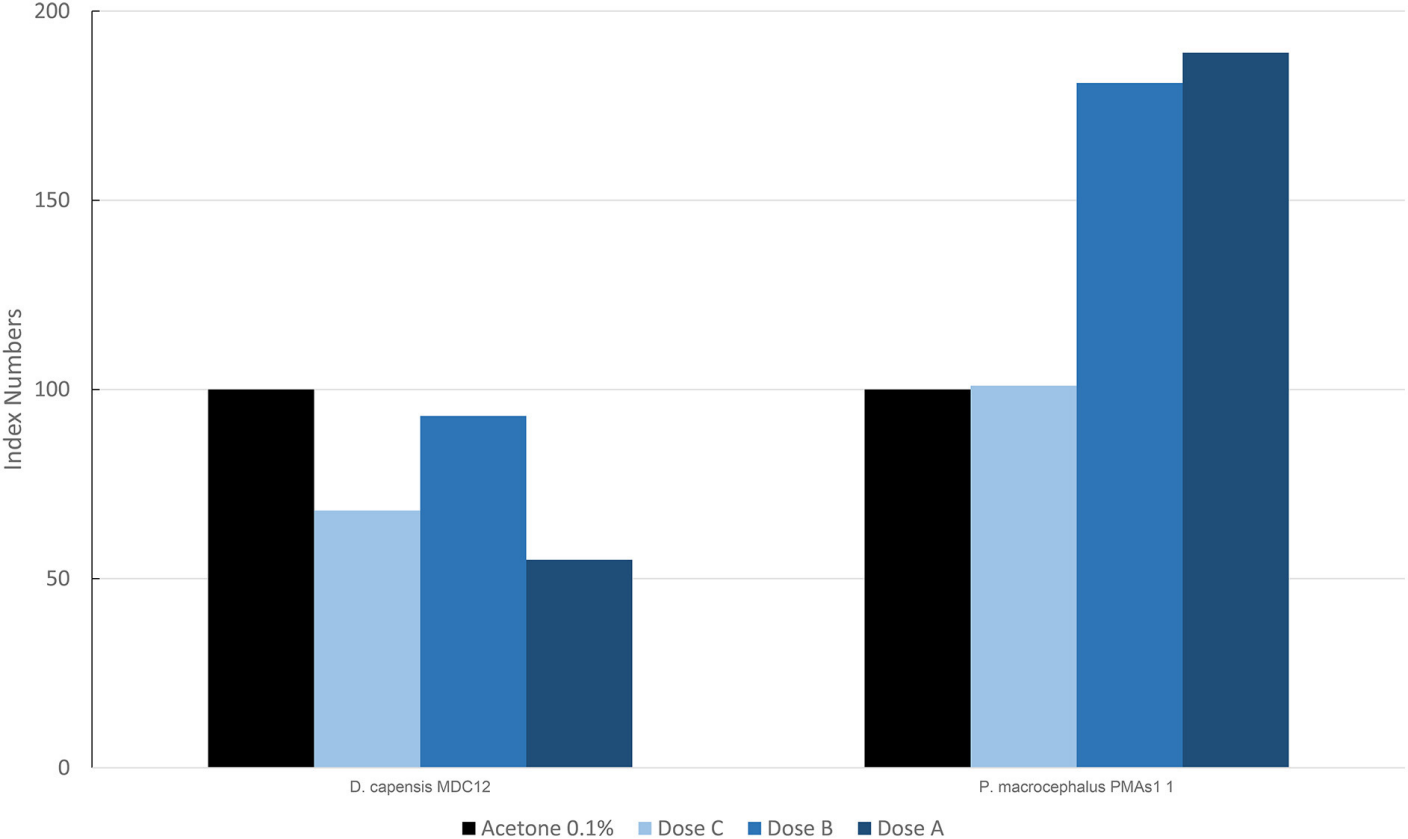
Mean values of immunofluorescence of MICA expressed as index numbers respect to the solvent control (acetone) revealed in cultured fibroblasts of different cetacean species treated with PAHs.

In Figure 5, the results are reported for the mean levels of immunofluorescence of MICA, which was detected in cultured fibroblasts of different species treated with PAHs and expressed as index numbers relative to the solvent control (acetone). Only two species, the long-beaked common dolphin and the sperm whale (PMAS1), were exposed to PAHs. In the sperm whale, a significant increase was present in the level of MICA, with a trend in the dose/response type. The long-beaked common dolphin showed a discontinuous response but overall a downregulation.

**Figure 6:**
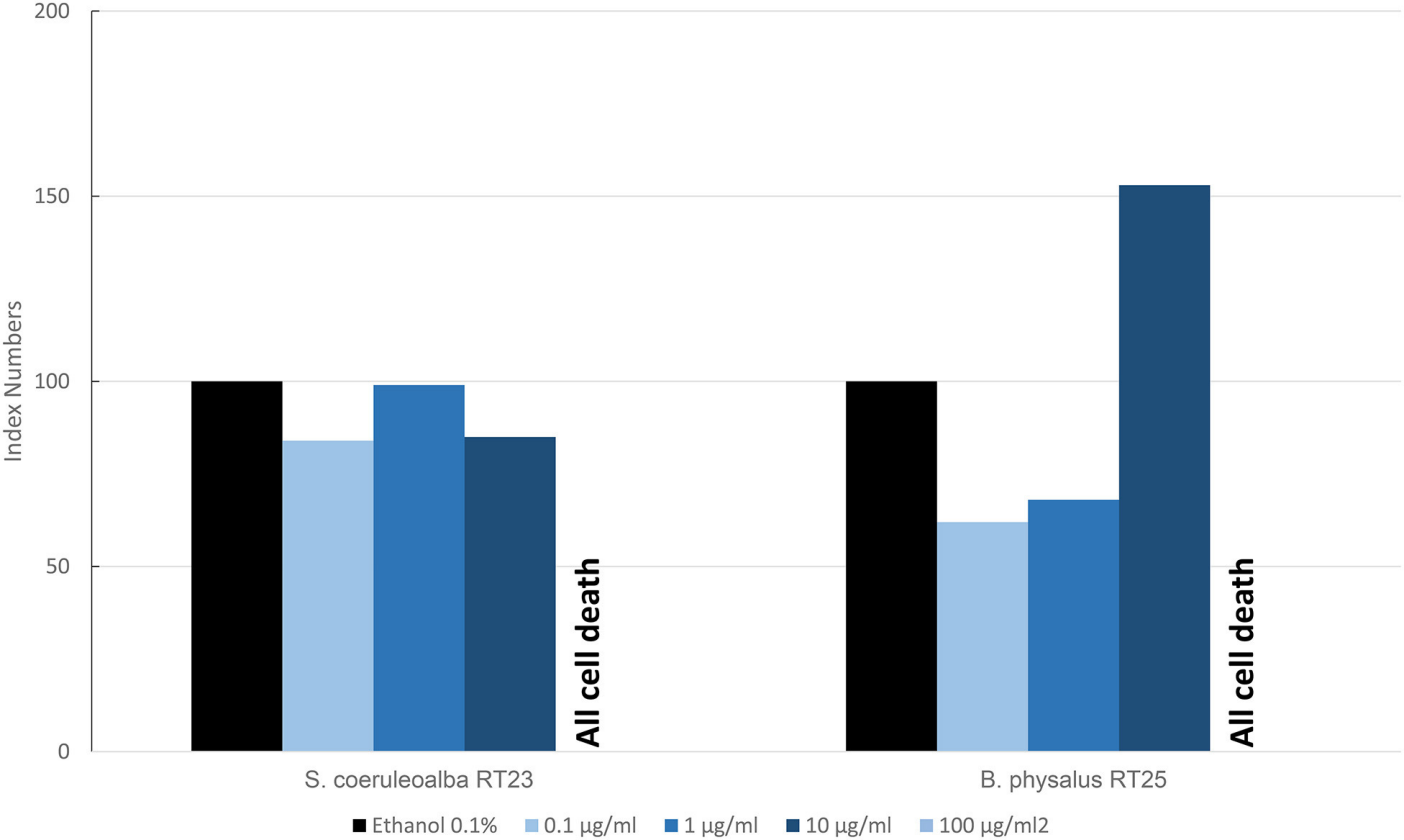
Mean values of immunofluorescence of MICA expressed as index numbers respect to the solvent control (ethanol) revealed in cultured fibroblasts of different cetacean species treated with BPA.

Figure 6 shows the results of the mean levels of immunofluorescence of MICA, found in cultured fibroblasts of different cetacean species treated with BPA and expressed as index numbers relative to the solvent control (ethanol). We investigated only two specimens: a fin whale and a striped dolphin. Their responses were markedly different, even if the higher dose caused the death of all cells in both animals, thus highlighting its toxicity. Only the fin whale fibroblasts exhibited an upregulation phenomenon compared to the solvent control.

**Figure 7:**
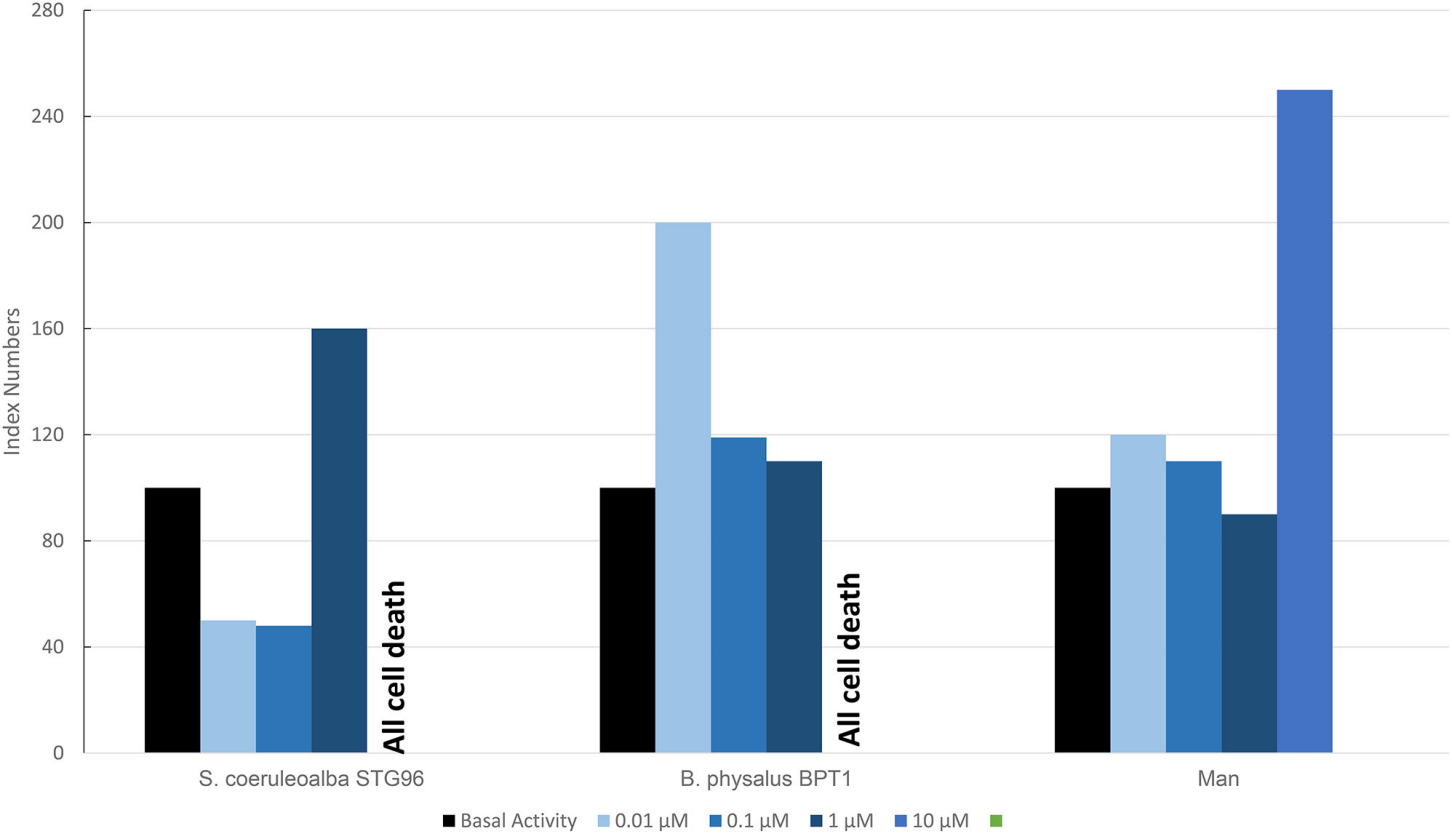
Mean values of immunofluorescence of MICA expressed as index numbers respect to basal activity revealed in cultured fibroblasts of different cetacean species and in man treated with MeHg.

Figure 7 shows results of the mean levels of immunofluorescence of MICA, found in cultured fibroblasts of different cetacean species and man treated with MeHg and expressed as index numbers with respect to the blank and represented by cells treated only with primary and secondary antibodies. Although the responses were different, in the two cetacean species compared to human skin fibroblasts, at least one dose of treatment with MeHg caused upregulation of MICA. Additionally, in this case, as for PAHs in cetaceans, the higher dose caused the death of all cells, while in man with this dose, an evident upregulation of MICA was documented.

### Summary of Treatment Results

What we can summarize from all the herein presented results is shown in Table 2, where for each specimen, the results of upregulation, downregulation, or no MICA response, following a specific treatment, are reported.

**Table 2:** Summary of response types with different treatments in different specimens.

	**OCs**	**PBDEs**	**PAHs**	**BPA**	**MeHg**
*Orcinus orca MOO12*	↓	↓	n.t.	n.t.	n.t.
*Delphinus capensis MDC12*	↓	↑	↓	n.t.	n.t.
*Balaenoptera edeni MBE3*	↑	↓	n.t.	n.t.	n.t.
*Physeter macrocephalus PMAs1*	↑	↓	↑	n.t.	n.t.
*Physeter macrocephalus PMAs2*	↑	↑	n.t.	n.t.	n.t.
*Stenella coeruleoalba RT23*	↓	↑	n.t.	-	n.t.
*Stenella coeruleoalba RT17*	↓	↑	n.t.	n.t.	n.t.
*Stenella coeruleoalba STG96*	n.t.	n.t.	n.t.	n.t.	↑
*Balaenoptera physalus RT25*	-	↑	n.t.	↑	n.t.
*Balaenoptera physalus BPT1*	-	n.t.	n.t.	n.t.	↑
*Man*	↑	n.t.	n.t.	n.t.	↑

*Different color of box is related to different response of MICA: orange, upregulation; green, downregulation; yellow, no alteration; n.t., no treatment*.

Some specimens had the same response patterns even with different treatments, such as the killer whale (MOO12), which always underwent a downregulation with both OCs and PBDEs. In both cases, the differences between the carrier and the doses were statistically significant with the non-parametric Kruskal-Wallis test (*p* < 0.05). The long-beaked common dolphin (MDC12) showed a downregulation with both OCs and PAHs, while simultaneously exhibiting an upregulation with PBDEs. Again, these responses were statistically significant. The Bryde's whale (MBE3) showed a MICA upregulation with OCs and a downregulation with PBDEs, both statistically significant. In the two specimens of sperm whale, the predominant response was an upregulation. Downregulation occurred in PMAs1 only for the PBDE treatment, but it was not statistically significant, unlike all others, both in PMAs1 and in PMAs2. RT23 and RT17 specimens of striped dolphins showed the same MICA behavior after treatments, with downregulation with PCBs and upregulation with PBDEs. Whereas, with PCBs, the reaction was statistically significant in both samples, for the PBDEs, it was not. With BPA in the RT23 striped dolphin, we did not observe a different response between the carrier and the doses. The STG96 striped dolphin treated with MeHg showed a statistically significant upregulation. The two specimens of fin whales (RT25 and BPT1) treated with OCs did not show a change in MICA levels between the carrier and the doses, whereas they constantly exhibited a statistically significant upregulation with the other treatments. Finally, in human fibroblast cell cultures, taken as a comparative study model, MICA upregulation occurred with OCs and with MeHg and, in both cases, it was statistically significant.

## Conclusions

In conclusion, we can state that MICA expression has been extensively investigated herein and validated as an important immune response biomarker in cetaceans, with the possibility of being also evaluated in skin biopsies from free-ranging individuals, when it is not possible to have blood as the biological investigation material. The response of upregulation or downregulation in the presence of some of the main environmental contaminants needs to be explored further, both from an ecological and biological standpoint. In fact, factors such as the area in which cetaceans live, coupled with their position(s) within the marine food webs as well as their sex, age, and their general health condition(s) can strongly influence MICA expression patterns. However, equally evident is that MICA expression modifications/alterations occur both with an increase or with a decrease in ecotoxicological stress levels, indicating either a “stress condition” or an immune response alteration/modification. The main result of this study refers, therefore, to “ecotoxicological stress-associated MICA expression's modulation,” and this is particularly interesting because these marine mammals are the main accumulators of liposoluble and persistent contaminants. If the accumulation of these substances joins the presence of infectious pathogens, such as DMV or *T. gondii*, these exposures and their deleterious effects could be potentially amplified, making the synergy of the two phenomena inseparable in the evaluation of the conservation status of these highly threatened and vulnerable species.

## Ethics Statement

The cetaceans tissues collection procedure was carried out in strict accordance with the relevant national and international guidelines under CITES permits (CITES Nat. IT025IS, Int. CITES IT 007).

## Author Contributions

LM designed and provided the resources for the study and performed the sampling, in *vitro* experiments and laboratory analysis and also elaborated the data and wrote, reviewed, and edited the manuscript. GDG and SM provided scientific support in the interpretation of lab data and they also revised and critically reviewed the manuscript. SC collaborated in performing the *in vitro* experiments, lab investigations, and data analysis and also wrote and reviewed the manuscript.

### Conflict of Interest Statement

The authors declare that the research was conducted in the absence of any commercial or financial relationships that could be construed as a potential conflict of interest.
